# GSDME-dependent pyroptosis signaling pathway in diabetic nephropathy

**DOI:** 10.1038/s41420-023-01452-8

**Published:** 2023-05-11

**Authors:** Shengyu Li, Lifeng Feng, Guangru Li, Ruiqing Liu, Changzhen Ma, Lin Wang, Aijiao Gao, Chang Liu, Yujie Cui, Zecheng Jiang, Yuhang Xie, Qiang Wu, Xia Wang, Liang Yang, Zhi Qi, Yanna Shen

**Affiliations:** 1 School of Medical Laboratory, Tianjin Medical University, 300203 Tianjin, China; 2 Department of Molecular Pharmacology, School of Medicine, Nankai University, 300071 Tianjin, China; 3 Key Laboratory of Emergency and Trauma of Ministry of Education, Hainan Medical University, 571199 Haikou, China; 4 Department of Laboratory Medicine, West China Second University Hospital, Sichuan University; key laboratory of birth defects and related diseases of women and children (Sichuan University), Ministry of Education, 610041 Chengdu, Sichuan China; 5 Tianjin Key Laboratory of General Surgery in Construction, Tianjin Union Medical Center, 300121 Tianjin, China; 6 Xinjiang Production and Construction Corps Hospital, 830092 Xinjiang, China

**Keywords:** Molecular biology, Diseases

## Abstract

Diabetic nephropathy (DN) is one of the serious chronic microvascular complications of diabetes, and leads to the increased morbidity and mortality in diabetic patients. Gasdermin E (GSDME)-dependent pyroptosis signaling pathway plays important roles in a variety of physiological and pathological processes. However, its role and mechanism in DN are still unclear. In this study, we established a rat DN model by intraperitoneal injection of streptozotocin (STZ) successfully. Structural and functional disorders in the kidney were exhibited on the 12th week after STZ injection; the expressions of caspase-3 and GSDME at protein level in renal cortex were significantly up-regulated. At the 20th week, GSDME-N increased significantly, accompanied by the upregulation of caspase-1 in renal cortex and the release of mature IL-1β (mIL-1β) in serum. Furthermore, we found the protein levels of GSDME, caspase-3, caspase-1 and IL-1β were all increased in HK2 and HBZY-1 cells under high-glucose conditions. We also found that the expression of GSDME-N significantly decreased when caspase-3 was knockdown. In contrast, knockdown of GSDME has no effect on caspase-3. Interestingly, either caspase-3, caspase-1 or GSDME knockdown reduced the release of mIL-1β. Finally, injection of adeno-associated virus (AAV) 9-shGSDME into the rat kidney reduced kidney damage and renal cell pyroptosis in comparison with wild-type diabetic rats. These results indicated that the activation of caspase-1 induced IL-1β maturation, and the activation of caspase-3 mediated cleavage of GSDME responsible for the formation of plasma membrane pore, followed by cytoplasmic release of mIL-1β. Overall, we identified a pro-pyroptosis role for GSDME in DN, which does provide an important basis for clinical therapeutic studies.

## Introduction

Diabetic nephropathy (DN), one of the serious chronic microvascular complications of diabetes, is the main cause of end-stage renal disease (ESRD) with increased morbidity and mortality in diabetic patients [1–4]. Accumulating evidences showed that many critical biological processes were involved in DN, including inflammation, renal interstitial fibrosis, oxidative stress and so on [5–8]. Besides, numerous studies have shown that cell death was thought to contribute to progressive kidney cell injury and cell depletion in DN [9–11]. However, the detailed mechanism of cell death involved in DN is poorly understood. Thence, the exploration of exact molecular mechanisms related to the development of DN is urgently needed.

Pyroptosis is an inflammatory form of caspase-dependent programmed cell death (PCD) in eukaryotic cells [12, 13]. It is characterized by cell swelling and plasma membrane large bubbles blowing, pore-induced intracellular traps (PITs) forming plasma membrane rupturing and pro-inflammatory intracellular contents releasing (such as mature IL-1β) [12–14]. The released intracellular contents attract more immune cells and trigger local inflammation, thereby strengthening the immune defense function of cells [14, 15]. However, recent papers have reported that pyroptosis accompanied by a massive expression of pro-inflammatory mediators were related to the progression of diabetes as well. Qiu et al. suggested that hyperglycemia-induced pyroptosis aggravated myocardial ischemia/reperfusion injury in diabetic rats [16]. Che et al. have also confirmed that high glucose caused pyroptosis of neurons [17]. While few studies focused on the participation of pyroptosis in the pathogenesis of DN.

Gasdermin (GSDM) is a family of proteins (6 in humans: GSDMA, GSDMB, GSDMC, GSDMD, GSDME, DFNB59) that share about 45% sequence homology [14]. All of the GSDM (except for DFNB59) adopt a two-domain architecture that is separated into the presumed gasdermin-N and gasdermin-C domains with a long loop harboring the inflammatory caspase cleavage site. The gasdermin-N domain is the most conserved region, which locates to the mammalian cell membrane to form pores. Along with the release of mIL-1β, the whole process is defined as gasdermin-mediated PCD—pyroptosis [18, 19]. At present, there are many studies on GSDMD-dependent pyroptosis signal pathways in the heart [20], kidney [21], tumor [22] and other diseases. Gasdermin E (GSDME), originally named deafness autosomal dominant 5 (DFNA5), leads to deafness characterized by an autosomal dominant inheritance and gradual loss of hearing, as a result of outer hair cells apoptosis [23]. Recently, many studies have shown that GSDME plays an essential role in promoting tumor cell pyroptosis induced by chemotherapeutic drugs. Wang et al. showed that GSDME switched caspase-3-mediated apoptosis induced by TNF or chemotherapy drugs to pyroptosis [24]. An et al. showed that tetraarsenic hexoxide enhanced the generation of mitochondrial ROS to promote pyroptosis by inducing the activation of caspase-3/GSDME in triple-negative breast cancer cells [25]. Zhang et al. showed that miltirone induces cell death in hepatocellular carcinoma cells through GSDME-dependent pyroptosis. Current research has found that GSDME was highly expressed in a variety of organs, such as lung, kidney, testis, and placenta; it is also moderately expressed in the heart, pancreas, stomach, small intestines, and brain [26–28]. Zheng et al. also found that GSDME promoted the pyroptosis of cardiomyocyte cells [29]. However, little is known about the role of GSDME in kidney disease, especially in DN. Interestingly, we found increased expression of GSDME in kidneys of DN rats in our current study. Herein, we investigate whether GSDME-dependent pyroptosis play a critical role in the development of DN.

## Results

### Biological characteristics and kidney morphological changes in DN rats

**Fig. 1:**
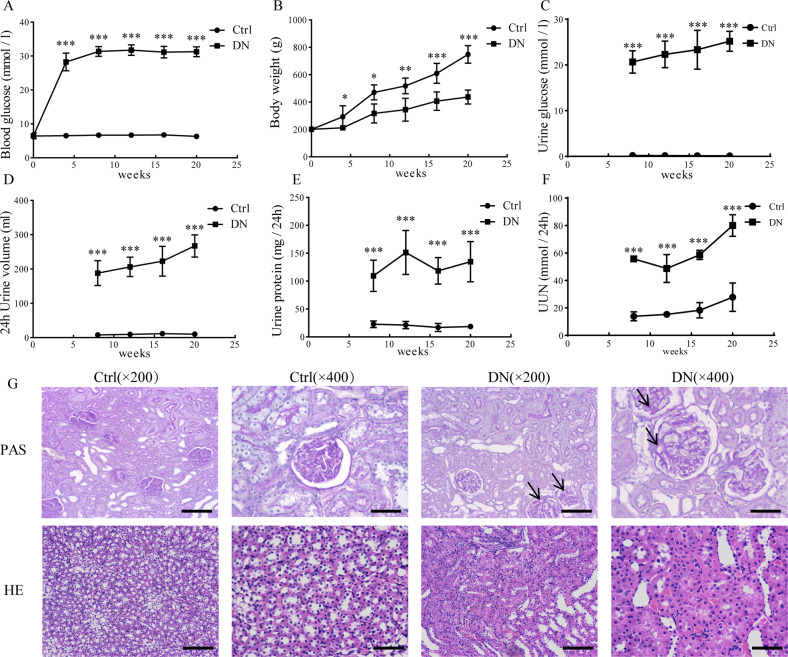
Biological characteristics and kidney morphological changes in rat models of DN. **A**, **B** Non-fasting blood glucose and body weight at 0, 4, 8, 12, 16, and 20 weeks. **C**–**F** Urine glucose, 24 h urine volume, 24 h urine protein, and 24 h UUN levels at the 8, 12, 16, and 20 weeks. **G**. PAS staining and HE staining of renal cortex at 12 weeks. The arrowheads represent the sections of expanding glomerular basement membrane. Scale bar: 100 μm (×200), scale bar: 50 μm (×400). *P < 0.05, **P < 0.01, ***P < 0.001 vs. Ctrl group. Ctrl group: *n* = 5, DN group: *n* = 6.

DN rats presented higher level of non-fasting blood glucose (25.6–32.7 mmol/l), while rats in the Ctrl group maintained non-fasting blood glucose at a normal level (Fig. 1A). Body weight in DN group were significantly decreased when compared with those in the Ctrl group (Fig. 1B). Meanwhile, rats in DN group exhibited noticeably higher urine glucose, 24 h urine volume, 24-h urine protein, and 24-h urine urea nitrogen (UUN) in comparison with Ctrl rats at 8, 12, 16, and 20 weeks, respectively (Fig. 1C–F). Furthermore, PAS staining of the renal cortex at 12 weeks revealed mesangial expansion, focal tubular hypertrophy, and extracellular matrix accumulation in DN rats’ kidney cortex; in contrast, these alterations were not observed in the Ctrl group. HE staining showed significant tubular hypertrophy and tubule-interstitial injury in DN rats, whereas no hypertrophy and interstitial injury in the Ctrl group (Fig. 1G). The above results indicated that the kidney structure and function of diabetic rats were disordered, and the DN model was successfully established in rats.

### Activation of the GSDME signaling pathway in DN rats

**Fig. 2:**
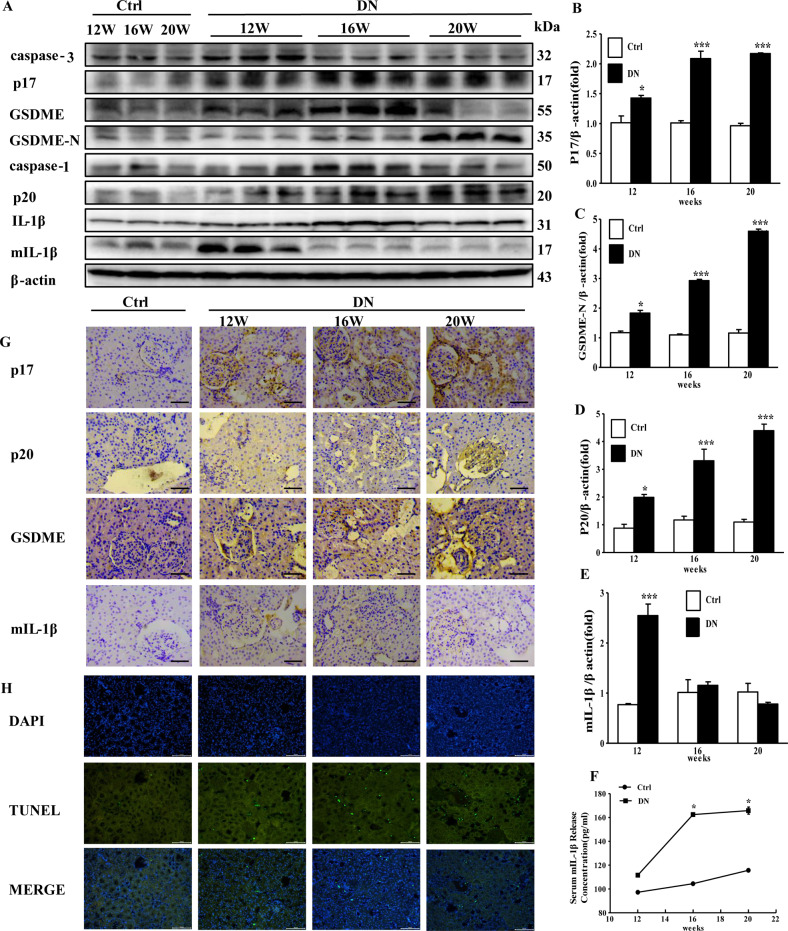
GSDME-dependent pyroptosis signaling pathway is activated in DN rats. **A** Western blotting analysis of caspase-3, p17, GSDME, GSDME-N, caspase-1, p20, IL-1β, and mIL-1β in kidney cortex of the DN and Ctrl group at 12, 16, and 20 weeks (*n* = 3 per group). **B**–**E** Quantification analysis of p17, GSDME-N, p20 and mIL-1β in DN and Ctrl group (*n* = 3 per group). **F** The mIL-1β concentrations in serum at 12, 16, and 20 weeks (*n* = 5 per group). **G** Immunohistochemistry staining of caspase-3 p17, caspase-1 p20, GSDME and mIL-1β in Ctrl and DN rats at 12, 16 and 20 weeks (scale bar: 50 µm). **H** TUNEL staining was performed in the kidney cortex for each group (scale bar: 100 µm). *P < 0.05, **P < 0.01, ***P < 0.001 vs. Ctrl rats.

To further investigate the molecular mechanism of renal injury in DN, we examined the effects of the GSDME pathway in the rat DN model. Western blotting analysis was used to determine the protein levels of GSDME, caspase-3, caspase-1, and IL-1β in rats’ kidney cortex at 12, 16, and 20 weeks. We found that the protein levels of GSDME-N, cleaved caspase-3 (p17), and cleaved caspase-1 (p20) were significantly increased in the DN group in a time-dependent manner. The protein levels of mature IL-1β (mIL-1β) were significantly increased at 12 weeks in DN group which in compassion with Ctrl group, and then decreased at 16 weeks and 20 weeks in DN group (Fig. 2A–E). Furthermore, the mIL-1β concentrations in the serum of DN group markedly increased at 16 weeks and 20 weeks compared with those in the Ctrl groups (Fig. 2F). Consistently, immunohistochemical staining showed that the protein levels of p17, p20, and GSDME were increased obviously in the kidney cortex of DN rats compared with those in Ctrl group. Meanwhile, the expression of mIL-1β was significantly increased at 12 weeks in comparison with that in the Ctrl group and then decreased in a time-dependent manner in the DN group (Fig. 2G). Besides, TUNEL staining was performed in the kidney cortex, the number of TUNEL-positive cells in DN group were significantly increased at 20 weeks compared with that in the Ctrl group (Fig. 2H). Taken together, our data strongly demonstrated that the GSDME signaling pathway played a critical role in the development of DN.

### High-glucose-induced pyroptosis in HK2 and HBZY-1 cells

**Fig. 3:**
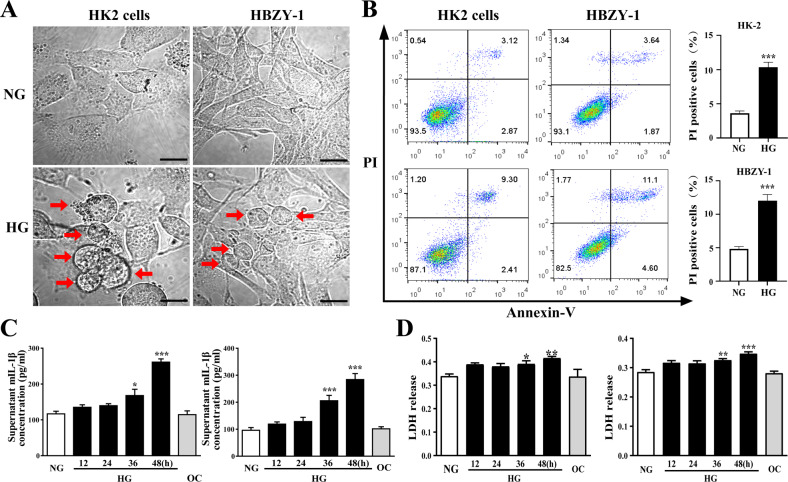
High-glucose induced pyroptosis in HK2 and HBZY-1 cells. **A** Phase-contrast imaging assay of HK2 and HBZY-1 cells cultured in normal glucose condition (NG, 5.5 mM) or high-glucose condition (HG, 25 mM) for 48 h. The arrowheads showed obvious swelling characteristic large bubbles on the plasma membrane of cells (scale bar, 5 μm). **B** Flow cytometry analysis of HK2 and HBZY-1 cells stained by annexin-V and propidium iodide (PI). Statistical analysis results of the percentage of PI ^+^ cells. **C** The mIL-1β concentration in supernatant of HK2 and HBZY-1 cells treated with NG, HG or OC for 0–48 h. **D** LDH release from HK2 and HBZY-1 cells treated with NG, HG, or OC for 48 h. *P < 0.05, **P < 0.01, ***P < 0.001 vs. NG group.

In order to further explore the underlying mechanism for pyroptosis in DN, an in vitro experiment was performed. We cultured two kinds of renal cells (proximal tubule epithelial cells (HK2) and rat glomerular mesangial cells (HBZY-1)) in different glucose concentrations (normal glucose: NG, 5.5 mM glucose or high glucose: HG, 25 mM glucose) for 48 h. Both HK2 and HBZY-1 cells cultured in HG condition showed obvious plasma membrane swelling under a phase-contrast microscope. However, this phenomenon was not observed in the NG group (Fig. 3A). Moreover, flow cytometry analysis using annexin-V and propidium iodide (PI) staining quantified the PI-positive cells. The results were consistent with the phase-contrast imaging assay, high glucose significantly increased the ratio of PI-positive HK2 and HBZY-1 cells compared with the normal glucose group. (Fig. 3B). Further, we found that the high glucose caused higher mIL-1β and LDH release in the supernatant of HK2 and HBZY-1 cells in comparison with the NG group, especially at 48 h (Fig. 3C, D). By contrast, the mannitol (isotonic control (OG)) group had no effect on the release of mIL-1β and LDH in the supernatant of HK2 and HBZY-1 cells (Fig. 3C, D). These findings suggested that high-glucose culture could induce pyroptosis in HK2 and HBZY-1 cells.

### The upregulation of pyroptosis-related proteins in HK2 and HBZY-1 cells under high-glucose conditions

**Fig. 4:**
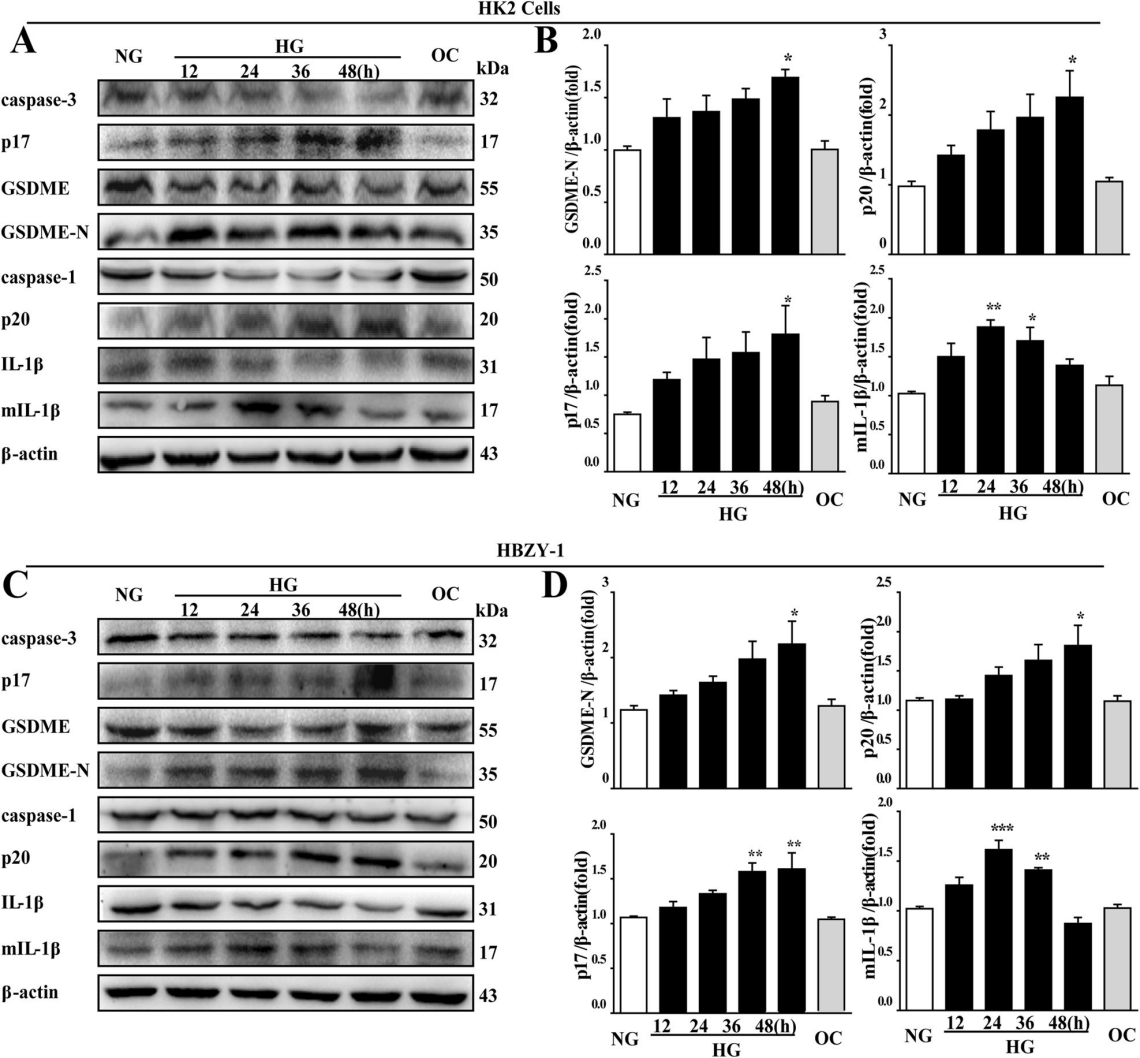
The protein levels of GSDME, caspase-3 and caspase-1, IL-1β were altered in HK2 and HBZY-1 cells under high-glucose conditions. **A** Western blotting analysis of caspase-3, p17, GSDME, GSDME-N, caspase-1, p20, IL-1β, and mIL-1β of HK2 cells. **B** Relative quantification analysis of GSDME-N, p17, p20, and mIL-1β in HK2 cells**. C** Western blotting analysis of caspase-3, p17, GSDME, GSDME-N, caspase-1, p20, IL-1β, and mIL-1β of HBZY-1. **D** Relative quantification analysis of GSDME-N, p17, p20, and mIL-1β in HBZY-1 cells. Data were presented as the mean ± SEM of the mean from three independent experiments. *P < 0.05, **P < 0.01, ***P < 0.001 vs. NG group.

Next, in order to further explore the effect of high-glucose culture on pyroptosis-related proteins in renal cell lines in vitro, western blotting analysis was used to determine the protein expressions of the active form of GSDME, caspase-3, caspase-1, and IL-1β in HK2 and HBZY-1 cells. As shown in Fig. 4, the protein expression levels of GSDME-N, p17, and p20 were significantly increased in the HG group compared with those in the NG group at 48 h. Whereas the mIL-1β protein levels in both cells were significantly increased at 24 h in comparison with the NG group and then decreased in a time-dependent manner in the HG group (Fig. 4B, D). In summary, high glucose induced the activation of the pyroptosis signaling pathway in HK2 and HBZY-1 cells in vitro.

### Attenuation of pyroptosis by inhibition of GSDME signaling pathway in HK2 and HBZY-1 cells

**Fig. 5:**
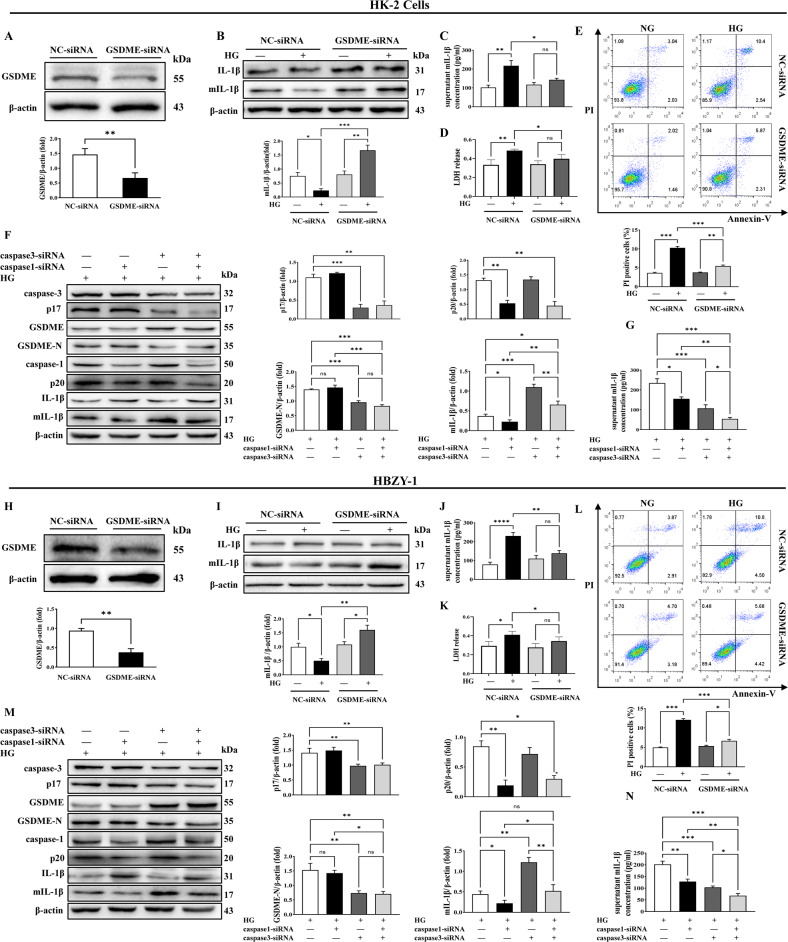
Caspase-3/GSDME signal pathway played a critical role in regulating high-glucose-induced pyroptosis in HK2 and HBZY-1 cells. **A**, **H** Western blotting analysis and relative densitometric quantification analysis of GSDME in HK2 and HBZY-1 cells transfected with GSDME-siRNA. **B**, **I** The protein expression levels and their corresponding relative densitometric quantification analysis of IL-1β and mIL-1β in HK2 and HBZY-1 cells under NG or HG conditions with or without GSDME-siRNA treatment. **C**, **J** The mIL-1β concentrations in the culture supernatant of HK2 and HBZY-1 cells transfected with or without GSDME-siRNA treatment. **D**, **K** The LDH concentrations in the culture supernatant of HK2 and HBZY-1 cells under NG or HG conditions with or without GSDME-siRNA treatment. **E**, **L** Percentage of PI^+^ HK2 and HBZY-1 cells were detected by flow cytometry under NG or HG conditions with or without GSDME-siRNA treatment. **F**, **M** The protein expression levels as well as densitometry analysis of caspase-3, p17, GSDME, GSDME-N, caspase-1, p20, IL-1β, and mIL-1β in HK2 and HBZY-1 cells transfected with caspase-1-siRNA, caspase-3-siRNA or both under HG conditions for 48 h. **G**, **N** The mIL-1β concentrations in the culture supernatant of HK2 and HBZY-1 cells transfected with caspase-1-siRNA, caspase-3-siRNA, or both under HG conditions for 48 h were detected by ELISA. The data were presented as the mean ± SEM of three independent experiments. *P < 0.05, **P < 0.01, ***P < 0.001. ns: not significant.

In order to further confirm the critical role of GSDME in high-glucose-induced pyroptosis in HK2 and HBZY-1 cells, both cells were transfected with GSDME-siRNA, the GSDME protein level was significantly decreased in comparison with that in cells transfected with NC-siRNA (Fig. 5A, H). Moreover, compared with cells transfected with NC-siRNA, the protein level of mIL-1β in both cells transfected with GSDME-siRNA were significantly increased under HG condition (Fig. 5B, I). In contrary, the release of mIL-1β and LDH in the supernatant of both cells transfected with GSDME-siRNA were significantly reduced under HG condition (Fig. 5C, D, J, K), indicating that GSDME increased the release of mIL-1β and LDH from cells to the culture supernatant. Flow cytometry analysis indicated that the PI-positive HK2 and HBZY-1 cells were dramatically decreased when cells transfected with GSDME-siRNA under high-glucose conditions (Fig. 5E, L). Previous studies had revealed that caspase-3 activation specifically cleaved GSDME to induce pyroptosis in cancer cells [24]. In Fig. 5F, M, when cells transfected with caspase-3-siRNA, the protein expressions of caspase-3 and p17 in both cells were significantly reduced, GSDME-N but not GSDME in both cells were significantly reduced, indicating that cleavage of GSDME was mediated by caspase-3 activation. Furthermore, the expression of mIL-1β in cells was increased, whereas mIL-1β concentrations in the supernatant of both cells were significantly reduced when caspase-3 was knockdown (Fig. 5F, G, M, N), suggesting that cleavage of GSDME (GSDME-N) might be critical for the release of mIL-1β. Simultaneously, protein expressions of both caspase-1 and p20 in cells transfected with caspase-1-siRNA were significantly reduced. But the protein level of GSDME-N had no change (Fig. 5F, M). The expression of mIL-1β in cells as well as the mIL-1β concentration in the supernatant were obviously reduced (Fig. 5F, G, M, N), indicating that caspase-1 activation was essential for IL-1β expression and maturation, however it did not affect the perforation of GSDME-N in the cell membrane. When HK2 and HBZY-1 cells were transfected with caspase-3-siRNA and caspase-1-siRNA combination, the protein levels of GSDME-N, caspase-1, and p20, caspase-3 and p17 were markedly reduced (Fig. 5F, M). The protein level of mIL-1β in cells was much higher than those of no treatment group and caspase-1-siRNA group. In the contrary, the protein level of mIL-1β was significantly reduced compared with the caspase-3-siRNA group (Fig. 5F, M). The mIL-1β concentration in the supernatant of both cells was reduced compared with all the other groups (Fig. 5G, N). Taken together, the above data demonstrated that activation of caspase-1 played a key role in IL-1β maturation. More importantly, the activation of caspase-3 mediated cleavage of GSDME responsible for plasma membrane pore-forming, which induced pyroptosis to lead the release of mIL-1β from HK2 and HBZY-1 cells into the supernatant.

### Significant reduction of DN-induced renal injury by adeno-associated virus 9 mediated GSDME gene knockdown therapy

**Fig. 6:**
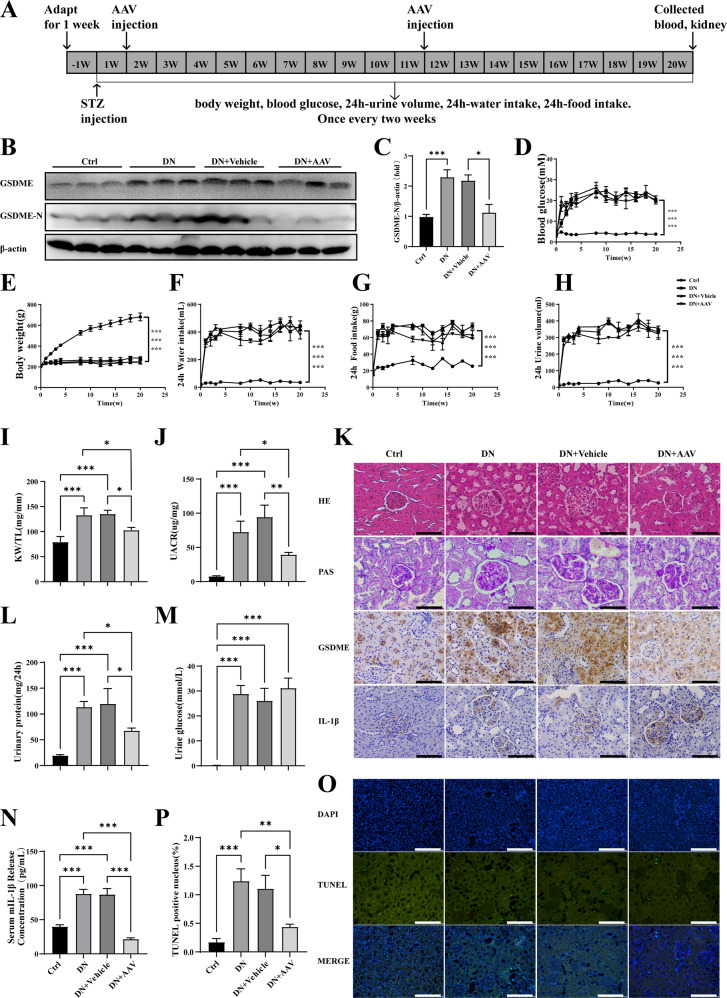
AAV9-shGSDME gene therapy alleviated kidney pyroptosis caused by DN. **A** Schematic diagram outlining the in vivo experiments. One week after adapting to the environment, SD rats were injected intraperitoneally with STZ to induce type 1 diabetes. First week and 11th week later, AAV9-shGSDME virus was injected into the renal cortex. Twenty weeks later, renal tissue and blood samples were collected. Body weight, 24-h urine volume, 24-h drinking water and 24-h diet were measured every two weeks. **B**, **C** Western blotting analysis of GSDME and GSDME-N in kidney cortex of the different group rats. **D**–**H** Fasting blood glucose levels, body weight, 24 h water intake, 24 h food intake and 24 h urine volume in Ctrl group, DN group, DN+Vehicle group and DN + AAV group. **I** Kidney weight/ Tibia length (KW/TL). **J** Urinary protein/ creatinine (UACR). **L** Urinary protein. **M** Urine glucose were measured at the 20th week. **K** HE staining, PAS staining, GSDME and IL-1β immunohistochemistry staining of renal cortex for different group rats at 20th week (scale bar: 50 µm). **N** The mIL-1β concentrations of serum in different group rats at 20th week. **O**, **P** TUNEL staining was performed in the kidney cortex for each group (scale bar: 100 µm). *P < 0.05, **P < 0.01, ***P < 0.001.

Next, in order to further evaluate the key role of GSDME in DN, we injected the Adeno-associated virus 9 (AAV9)-shGSDME into the kidneys of diabetic rats at 1st week and 11th week after STZ injection. The blood and kidney tissues of rats were collected at the 20th week for detection. The fasting blood glucose, body weight, 24 h urine volume, 24 h food intake, and 24 h water intake of the rats were measured every two weeks (Fig. 6A). As shown in Fig. 6B, C, AAV9-mediated GSDME knockdown significantly decreased the protein expression of GSDME-N in the DN + AAV group in comparison with the DN+Vehicle group. Similarly, the immunohistochemical staining of the kidney showed lower expression of GSDME in the DN + AAV group as well (Fig. 6K). Interestingly, knockdown of GSDME had no effect on blood glucose levels in rats (Fig. 6D). The body weight of rats in the DN group was significantly lower than that in the Ctrl group, while there were no significant differences between DN, DN+Vehicle group, and DN + AAV group (Fig. 6E). Consistently, there was also no difference in 24 h water intake (Fig. 6F), 24 h food intake (Fig. 6G) and 24 h urine volume (Fig. 6H) between the DN+Vehicle group and the DN + AAV group. Furthermore, we examined the ratio of kidney weight to tibia length (KW/TL), urinary albumin creatinine ratio (UACR), 24 h urinary protein content, and urinary glucose concentration. We found that the knockdown of GSDME significantly reduced the ratio of KW/TL (Fig. 6I), UACR (Fig. 6J), and urinary protein (Fig. 6L). However, consistent with the trend in blood glucose level, there was no alteration in the concentration of urine glucose as well (Fig. 6M). HE staining also showed that the degree of tubular hypertrophy and tubule-interstitial injury in the DN + AAV group was significantly alleviated in comparison with that in DN and DN+Vehicle group (Fig. 6K). PAS staining revealed mesangial expansion, focal tubular hypertrophy, and extracellular matrix accumulation in the kidney cortex of DN+Vehicle rats, and these changes have been significantly improved after the GSDME knockdown (Fig. 6K). In addition, TUNEL staining was performed in the kidney cortex, and the number of TUNEL-positive cells in the DN + AAV group was significantly decreased at the 20th week compared with the DN+Vehicle group (Fig. 6O, P). Interestingly, after GSDME knockdown, the content of mIL-1β released into serum decreased significantly (Fig. 6N), but the content in the renal cortex increased (Fig. 6K), suggesting that the knockdown of GSDME affected the release of mIL-1β. As a result, the above data strongly demonstrated that AAV9-mediated GSDME gene knockdown significantly improved kidney damage caused by diabetes.

## Discussion

DN, characterized by proteinuria and progressive renal dysfunction, is one of the most common clinical complications of diabetes. High blood glucose acts as an independent risk factor [30]. The kidneys’ pathophysiological changes associated with DN include inflammatory cell infiltration, tubular and glomerular hypertrophy, mesangial expansion, fibrosis, extracellular matrix accumulation, cellular dysfunction, and eventually proteinuria [6, 31–33]. And the progressive loss of renal parenchymal cells plays a key role in the progression of DN [34–37]. Loss of renal parenchymal cells as a result of cell death is a response to the high-glucose environment. Among the different cell death forms, pyroptosis, a highly inflammatory form of PCD [38–40], is molecularly indefinite and urgently needed to be investigated. In our present study, blood glucose and body weight began to increase from the 4th week (Fig. 1A, B), indicating that the diabetes mellitus rat model was successfully established. Urine glucose, urine volume, urine protein and UUN were all increased from 8th week (Fig. 1C–F), however no morphological changes in the kidney were observed between Ctrl and DN group (data not shown). PAS staining and HE staining of the kidney cortex showed significant morphological changes in the kidney structure at 12th week in DN rats (Fig. 1G), indicating that the injury in the kidney widespread in the DN rats’ model. Similarly, Chen et al. demonstrated that the 12th week after STZ injection was the initial stage of DN [41]. The results of TUNEL staining (Fig. 2H), phase-contrast imaging assay (Fig. 3A) and flow cytometry (Fig. 3B) further confirmed that pyroptosis occurred both in vivo and in virto experiments, and it might contribute to the gradual loss of renal function in DN. Thus, we assumed that pyroptosis might play a crucial role in DN progression by acting as an important factor in the renal tubule and glomerular mesangial injury.

Recent study has shown that blocking pyroptosis by long noncoding RNA MALAT1 was effective in either slowing or reducing cell injury in models of DN [42]. In recent years, several studies have reported that GSDME belongs to a gasdermin family sharing about 45% sequence homology. It was mainly expressed in the placenta, brain, heart, kidney, cochlea, intestines, and IgE-primed mast cells. At present, some studies have found the promoting role of GSDME in renal disease and examined the molecular mechanism of pyroptosis, including nephrotoxicity induced by chemotherapy [26], obstructive renal disease [27] and acute renal injury [43]. However, few studies have been reported on the role of GSDME in DN. It is well known, GSDME is specifically cleaved by caspase-3 in its linker, generating a GSDME-N fragment that perforates membranes and thereby induces pyroptosis. In our study, the expression of mIL-1β in GSDME-siRNA-treated cells were significantly higher than that in NC-siRNA-treated cells in the presence of high glucose. Whereas the concentration of mIL-1β in the supernatant of GSDME-siRNA-treated cells markedly reduced when compared with NC-siRNA treatment group (Fig. 5B, C, I, J). Therefore, we speculated that GSDME-siRNA treatment retained most mIL-1β within cells and prevented the release of mIL-1β into the supernatant, indicating the dependence of mIL-1β release on GSDME activation. Similarly, Zhou et al. clarified that GSDME deletion increased the level of mIL-1β within the cells and inhibited the release of mIL-1β in THP-1 cells treated with nigericin, identifying GSDME as a conduit for mIL-1β release [44]. In addition, Feng et al. found that the knockdown of GSDME by siRNA significantly decreased inflammatory response by suppressing IL-1β expression and decreasing the release of mIL-1β in TNF-α-induced HUVECs [45]. Therefore, the regulatory effect of GSDME on IL-1β might be different in diverse diseases, which need more researches to further investigate the exact function of GSDME in different diseases.

The proteolytic enzymes called caspases have been investigated in several clinical disorders [46]. Caspases play crucial roles in the execution or final phase of cell death by cleaving and inactivating various structural and functional intracellular proteins which are essential for cell survival and proliferation. Evidence is now emerging to implicate the caspase signaling pathway in various renal diseases, including the pathogenesis of DN. Among the 14 known members of the caspase family thus far identified, several executioner caspases including caspases-3 may participate in the final degradation of intracellular proteins [47]. Caspase-3 plays an important role in apoptosis and pyroptosis pathways. Activation of caspase-3 cleaves a variety of downstream substrates that leads to the typical morphological and biochemical changes of apoptotic cells, including cell shrinkage, chromatin condensation, DNA fragmentation and the externalization of phosphatidylserine from the inner layer of the plasma membrane. When GSDME is highly expressed, the active caspase-3 cleaves GSDME to the N-terminal domain, which can execute pyroptosis by forming nonselective pores in the membrane, shifting the mode of cell death from apoptosis to pyroptosis [48–50]. Shen et al. suggested that activation of caspase-3 is associated with GSDME cleavage in cisplatin- or doxorubicin-treated renal tubular epithelial cells, and inhibition of caspase-3 alleviates the secretion of inflammatory cytokine and the deterioration of kidney function [26]. Li et al. found that deletion of caspase-3 or GSDME alleviated renal tubule damage and inflammation and finally prevented the development of hydronephrosis and kidney fibrosis after ureteral obstruction [27]. Zhang et al. proved that caspase-3-mediated GSDME induced pyroptosis in breast cancer cells. However, the role of caspase-3 in GSDME-mediated pyroptosis remains to be elucidated in DN [51]. In this study, upregulation of caspase-3 and its active form had been observed under high-glucose conditions simultaneously in vivo and in vitro.

Our data showed that caspase-3 p17 and caspase-1 p20 were significantly up-regulated in the kidneys of diabetic rats (Fig. 2A). Similar results were observed in HK2 and HBZY-1 cells exposed HG (Fig. 4A, C). Further, after silencing caspase-3 by siRNA, the down-regulation of cleavage of GSDME was accompanied by a decrease in pyroptosis followed by reducing in mIL-1β secretion (Fig. 5F, G, M, N). When cells were transfected with caspase-1-siRNA alone, the protein level of GSDME-N in cells was not different from that of NC-siRNA group under HG condition, whereas the protein level of mIL-1β in cells as well as the concentration of mIL-1β in the supernatant were obviously reduced (Fig. 5F, G, M, N). Thus, it was confirmed that GSDME was specifically cleaved by caspase-3, generating a GSDME-N fragment to form permeability pores on the plasma membrane for IL-1β secretion, then cause pyroptosis. While, in both caspase-3-siRNA and caspase-1-siRNA-treated HK2 and HBZY-1 cells, cleavage of GSDME, the protein level of mIL-1β in cell inclusion and concentration of mIL-1β in supernatant were entirely reduced (Fig. 5F, G, M, N). Interestingly, there was no superposition effect of caspase-3-siRNA and caspase-1-siRNA. Therefore, our research confirmed that caspase-3, but not caspase-1, played a leading role in the cleavage of GSDME-induced plasma membrane pore formation. Although caspase-1 cannot cleave GSDME, it can cleave IL-1β to form mature IL-1β, which was released through the pores formed by GSDME-N in the plasma membrane. This will provide a novel idea for research on pyroptosis in DN. Several studies showed that active caspase-1 induced to GSDMD cleavage and maturation of IL-1β, and then GSDMD-N caused pyroptosis by promoting the formation of membrane pores and the release of mIL-1β [22, 52]. To this point, we detected the expression of GSDMD in rats’ kidney tissue, our results showed that the protein level of GSDMD-N was significantly increased in DN group (Supplementary Fig. S1). However, when we used GSDME-siRNA in HK2 and HBZY-1 cells and used AAV9-GSDME knockdown virus in rats to inhibit the expression of GSDME, we discovered that knockdown of GSDME largely blocked the release of mIL-1β (Figs. 5C, J and 6N). Therefore, we speculate that GSDME-mediated pyroptosis plays a major role in regulating the mIL-1β release in DN.

Previous research has already revealed that inflammation is intrinsically link to the pathogenesis of DN [53–55]. The cellular mechanisms that modulate inflammatory signaling in DN are not clear yet. Pyroptosis is the process of pro-inflammatory PCD [14]. It can stimulate immune cells to release pro-inflammatory cytokines and undergo cell death in response to infection with intracellular pathogens or other dangerous signals. The released cytokines attract more immune cells, further perpetuating the inflammatory cascade in the tissue [55]. In this study, GSDME was cleaved after the activation of caspase-3 during pyroptosis and the inflammatory cytokines mIL-1β were converted and secreted following activation of caspase-1. mIL-1β was released through the pores formed by GSDME-N in the plasma membrane to cause inflammatory damage to the kidney. Those provide evidence for GSDME’s involvement in the inflammatory response to DN, and GSDME can also serve as a target for immunotherapy.

The role of GSDME-dependent pyroptosis in DN has been rarely reported, and we found that knockdown of GSDME reduced pyroptosis in renal cells and mIL-1β secretion in vitro, so we further validated it in in vivo experiments. In this study, we used an AAV9-packed GSDME knockdown virus, injected in situ in the kidney to provide maximum disruption efficiency, targeting, and safety [56]. Our results showed that GSDME was significantly reduced in the kidneys of rats with DN and was accompanied by recovery of renal function, demonstrating that knockdown of GSDME significantly improved the progression of DN (Fig. 6B–M). But surprisingly, the levels of urine glucose and blood glucose did not decrease significantly after the GSDME was knocked down (Fig. 6D, M), indicating that inhibition of GSDME attenuated injury of DN independed on glucose level. We speculated that knockdown of GSDME protects the kidney by reducing the pyroptosis of renal cells rather than blood glucose. Overall, although the expression of GSDME-N in kidney gradually increased with the progression of DN from 12th week to 20th week (Fig. 2A, C), the increase of GSDME further worsened the condition of DN (Fig. 6). Therefore, we assumed that GSDME-induced pyroptosis of renal cells might lead to the development of DN, and DN conversely accelerated GSDME-induced pyroptosis.

As we knew, GSDMD, a member of the gasdermin superfamily, was widely studied in pyroptosis. Several studies have revealed that GSDMD-mediated pyroptosis plays an important role in the development of DN in mice [21, 57, 58]. Importantly, Li et al. found that the expression of GSDMD was much higher in DN rats than the controls at 20 weeks post-DN [59]. To further examine which type of pyroptosis is more important in DN, we detected the protein levels of GSDMD and GSDMD-N in rats’ kidney cortex at 20 weeks post-DN. We found that the expression of GSDMD-N was significantly increased in DN group in comparison with Ctrl group, indicating that GSDMD-mediated pyroptosis may also play a role in the pathogenesis of DN (Supplementary Fig. S1). On the other hand, Current research has found that GSDME was highly expressed and play in important role in chemotherapy drug-induced nephrotoxicity as well as obstructive nephropathy [26, 27]. Little is known about the role of GSDME in DN. Therefore, we mainly investigated the role of GSDME-mediated pyroptosis in DN in this study. Surprisingly, in comparison with Ctrl group, we observed an approximately fourfold upregulation of GSDME-N protein levels (Fig. 2C), whereas GSDMD-N protein was only 1.4-fold upregulation in rats’ kidney cortex at 20 weeks post-DN (Supplementary Fig. S1). Although, which kind of pyroptosis is more important in DN needs to be further investigated in future work, above mentioned results indicated that GSDME-mediated pyroptosis may play a more important role in the pathogenesis of DN.

**Fig. 7:**
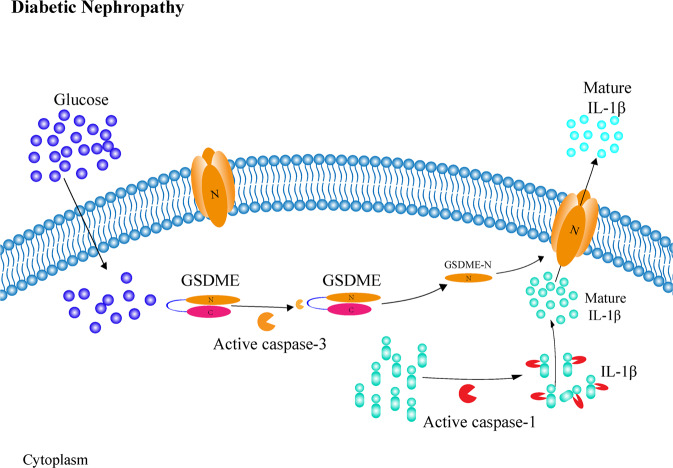
The cleavage of GSDME by caspase-3 induce pyroptosis in DN as a potential mechanism of renal injury. In conclusion, diabetes induced activation of caspase-1, which cleaved pro-IL-1β to mature IL-1β. Simultaneously, diabetes induced caspase-3 activation-dependent cleavage of GSDME. GSDME-N specifically binds to the plasma membrane to form pores, then inflammatory factors, such as mIL-1β released from cytoplasm, indicating GSDME-induced pyroptosis played a critical role in DN.

To our knowledge, this is the first time to report the mechanism that GSDME-dependent pyroptosis lead to renal injury and dysfunction in DN. In conclusion, pyroptosis is a form of inflammatory cell death. In this study, we confirmed that GSDME was cleaved by caspase-3, and GSDME-N fragments were generated to form permeability pores on the plasma membrane. The activated caspase-1 led to cleavage of pro-IL-1β to result in mature, bioactive mIL-1β. Subsequently, mIL-1β was released through the pores formed by GSDME-N in the plasma membrane to cause inflammatory damage in DN. Thus, the occurrence of pyroptosis is mediated by the synergistic action of the two pathways (Fig. 7). However, it is undeniable that the effect of GSDME on DN may be further investigated in GSDME^−^/^−^ rats and human kidney tissues. It may provide an effective therapeutic target associated with pyroptosis for DN.

## Materials and methods

### Reagents and antibodies

GSDME (ab215191), caspase-3 (ab13847), caspase-1 (ab179515), pro-IL-1β (ab2105) and mature (m)IL-1β (ab9722) antibodies were obtained from Abcam (Cambridge, UK); cleaved caspase-1 (3866), cleaved caspase-3(9661) antibodies were obtained from Cell Signaling Technology, Inc. (Danvers, MA, USA); β-actin (sc-47778) antibodies from Santa Cruz Biotechnology, Inc; anti-mouse IgG (H + L) HRP Conjugate (W402B) and anti-rabbit IgG (H + L) HRP (W401B) from Promega (Madison, WI, USA). All cell culture reagents were obtained from Thermo Fisher Scientific, Inc. TRIzol® reagent was obtained from Thermo Fisher Scientific, Inc. Lipofectamine® 3000 was purchased from Invitrogen (Thermo Fisher Scientific, Inc). Small interfering (si)RNA was purchased from Genepharma, Inc. Adeno-associated virus (AAV) 9-shGSDME virus purchased from HanBio Biotechnology Co. Ltd. (Shanghai, China). Short hairpin RNA (shRNA) sequences were designed by Hanbio Biotechnology to target rat GSDME.

### STZ-induced DN model

Sixty male Sprague–Dawley (SD) rats (180–220 g, 5-week-old) were purchased from the Laboratory Animal Center of the Academy of Military Medical Sciences (Beijing, China) and maintained under standard housing conditions of temperature (22 ± 4 °C) and humidity (60 ± 5%) with an alternating 12 h light/dark cycles and ad libitum access to a standard pellet diet and water throughout study. All rats were randomly divided into the diabetic nephropathy group (DN) and the normal group (Ctrl). DN rats (*n* = 36) received a single intraperitoneal (i.p.) injection of fresh streptozotocin (STZ; Sigma-Aldrich; Merck KGaA; 65 mg/kg in citrate buffer 0.1 mol/L, pH 4.5). Normal rats as control (*n* = 24) received an equal volume of sodium citrate buffer. The blood glucose levels were measured with a portable glucometer (UltraEasy, Johnson, USA) on day 3 and 7 after STZ or citrate buffer injection. Rats with non-fasting blood glucose concentrations over 16.7 mM in two consecutive determinations were defined as diabetic rats. Body weight, blood glucose, and urine samples (collected in metabolic cages at baseline) were monitored twice a week. Six DN rats and four control rats were sacrificed at 0, 4, 8, 12, 16, and 20 weeks, respectively, and the kidney and serum were collected for analysis. All the experimental procedures in the present study were performed in accordance with internationally recognized guidelines on animal welfare and approved by the Animal Care & Welfare Committee of Tianjin Medical University (Tianjin, China).

### AAV9-shGSDME injection

SD rats were anesthetized, and 1 × 10^12^ vector genome (vg)/ ml AAV9-shGSDME virus or 1 × 10^12^ vector genome (vg)/ ml AAV9-vehicle virus was injected into five different sites in the renal cortex (15μl/ site) in DN + AAV group or in DN+Vehicle group, respectively.

### Cell culture

Proximal tubule epithelial HK2 cells (cat. no. ZQ0313) and rat glomerular mesangial HBZY-1 cells (cat. no. ZQ0540) were purchased from Shanghai Zhong Qiao Xin Zhou Biotechnology Co., Ltd. (Shanghai, China). Cells were cultured in DMEM/F12 or DMEM at 37 °C and 5% CO_2_, supplemented with 10% FBS (Gibco, USA) and 100 units/ml penicillin-streptomycin (Sigma, St. Louis, MO), respectively. Cells plated on 60-mm dishes were cultured at a density of 1 × 10^6^ cells/well and treated with 5.5 mM (normal glucose group, NG) or 25 mM (high-glucose group, HG) or high mannitol (Mtol) concentration (5.5 mM glucose + 19.5 mM Mtol, OC) for 12, 24, 36 and 48 h.

### Transient transfection

As described in our previous article [60], cells were transfected with siRNA against caspase-1 (100 nM), caspase-3 (100 nM) and GSDME (100 nM), or their negative control (NC, 100 nM), respectively, using Lipofectamine® 3000 reagent following the manufacturer’s protocol. Sequences for caspase-1-hum-siRNA were as follows: sense, 5’-CCACUGAAAGAGUGACUUUTT-3’; antisense, 5’-AAAGUCACUCUUUCAGUGGTT-3’. Sequences for caspase-1-rat-siRNA were as follows: sense, 5’-GCAUUAAGAAGGCCCAUAUTT-3’; antisense, 5’-AUAUGGGCCUUCUUAAUGCTT’. Sequences for caspase-3-hum-siRNA were as follows: sense, 5’-GCAGCAAACCUCAGGGAAATT-3’; antisense, 5’-UUUCCCUGAGGUUUGCUGCTT-3’. sequences for caspase-3-rat-siRNA were as follows: sense, 5’-GCCGAAACUCUUCAUCAUUTT-3’; antisense, 5’-AAUGAUGAAGAGUUUCGGCTT-3’. Sequences for GSDME-hum-siRNA were as follows: sense, 5’-GGAGGUAGAUGUGCAGCAATT-3’; antisense, 5’-UUGCUGCACAUCUACCUCCTT-3’. Sequences for GSDME-rat-siRNA were as follows: sense, 5’-GCAAGUGUGAGAACCACAATT-3’; antisense, 5’-UUGUGGUUCUCACACUUGCTT-3’. Sequences for NC-siRNA as follows: sense, 5’-UUCUCCGAACGUGUCACGUTT-3’; antisense, 5’-ACGUGACACGUUCGGAGAATT-3’. After transfection, cells were incubated with normal (5.5 mm) or high (25 mm) glucose for 48 h and then collected for further experiments.

### Western blotting

Renal tissues were mechanically homogenized in modified RIPA lysis buffer (50 mmol/L Tris-HCl, pH 7.4; 1% Triton X-100; 0.25% Na-deoxycholate; 150 mmol/L NaCl; 1 mmol/L EDTA) supplemented with protease inhibitor (1:100 dilution, Sigma) and phosphatase inhibitor (1:100 dilution, Keygen Biotech. Inc). Proteins of HK2 cells and HBZY-1 cells were prepared using RIPA buffer (P0013B, Beyotime Biotechnology, China) supplemented with protease inhibitor and phosphatase inhibitor (1:100 dilution). After centrifugation (13,000 rpm at 4 °C for 30 min), supernatants were collected, and protein concentration was measured by the BCA protein assay kit (Pierce, Thermo). Protein samples from cell and kidney lysates (30 μg) were separated by electrophoresis on 10% SDS-polyacrylamide gels and transferred to a PVDF membrane (Millipore, USA). After blocking with 5% fat-free dry milk or BSA in Tris-buffered saline solution containing 0.05% Tween 20 for 2 h at room temperature and overnight incubation with specific primary antibodies (1:1000) at 4 °C, the membranes were rinsed with TBST buffer (0.1% Tween 20, 0.2 mM Tris, and 137 mM NaCl) and exposed to IgG-HRP-conjugated secondary antibody (1:5000) for 1 h at room temperature. Immunoreactive bands were visualized by enhanced chemiluminescent substrate (Millipore, USA). The bands were quantified densitometrically analyzed by Image J software 6.0 (National Institutes of Health, Bethesda, MD, USA) and normalized to the β-actin level.

### ELISA for measurement of IL-1β

The protein levels of IL-1β in rat serum or cultured supernatant were quantified using the commercial Rat Interleukin 1β (IL-1β) ELISA Kit (Bio-Swamp, Catalog Number: RA20020) and Human Interleukin 1β (IL-1β) ELISA Kit (Bio-Swamp, Catalog Number: HM10206), respectively, following the protocols from manufacturers.

### Histological examination

The renal cortex was fixed in 4% neutral buffered paraformaldehyde, embedded in paraffin and sectioned into 5-μm-thick sections. The sections were dewaxed and hydrated using standard sequential techniques at room temperature. Some sections were stained with hematoxylin and eosin (HE). The slides were mounted in a mounting medium (Solarbio, China) and observed under a light microscope (×200 and ×400; Nikon ECLIPSE-Ti). Some paraffin sections (5 µm) of the renal cortex were stained with 0.5% periodic acid-Schiff (PAS) for measurement of mesangial expansion and glomerulosclerosis injury. The stained sections were then viewed by a light microscope (200× and 400×; Nikon ECLIPSE-Ti).

### Immunohistochemistry staining

Sections were permeabilized with 1% Triton X-100 for 2 h and blocked with normal goat serum (Beyotime Institute of Biotechnology, Haiman, China) for 30 min at room temperature; the sections were incubated sequentially at 4 °C overnight with GSDME (1:500; ab175614; Abcam), caspase-3 (ab13847, 1:1500), caspase-1 (1:500; ab108362; Abcam) or mIL-1β (1 µg/ml; ab9722; Abcam) antibodies. Then incubated with mouse anti-rabbit IgG-HRP (sc-2357, 1:100) antibody for 2 h at room temperature. To visualize the signals, sections were treated with peroxidase substrate 3,3’-diaminobenzidine (DAB, 0.05%, ZSGB-Bio, China) and counterstained with hematoxylin for 1 min at room temperature. Sections were viewed and imaged under a light microscope (Ni-U; Nikon Corporation, Tokyo, Japan). Images were analyzed quantitatively using Image-Pro Plus 6.0 (Media Cybernetics, Inc., Rockville, MD, USA).

### TUNEL staining

TUNEL staining was performed with an In Situ Cell Death Detection kit (Roche Diagnostics GmbH) according to the manufacturer’s instructions. Sections were further incubated with TUNEL reaction mixture at 37 °C for 1 h in humid conditions. Coverslips were then washed in PBS and counterstained with DAPI (Sigma-Aldrich; Merck KGaA) at room temperature for 1 min. The slides were observed under the Nikon fluorescence microscope (Nikon Corporation; magnification, ×200).

### LDH release assay

LDH release was measured by the LDH-cytotoxicity assay kit (Nanjing Jiancheng Bioengineering Institute, China), according to the manufacturer. In brief, after HK2 and HBZY-1 cells were treated with high glucose for 48 h, the cell supernatant and cell lysate were collected, and the LDH content was detected according to the instructions. The LDH activity in the culture supernatant was expressed as a percentage of total LDH in the cell lysate.

### Statistical analysis

All data were presented as mean ± SEM (standard error of mean) with n representing the number of different experiments. Image J Acquisition and Analysis Software were used to analyze the western blotting results. The statistical significance of the differences between two groups was obtained by unpaired *t* tests. Differences among three or more groups were analyzed by one-way ANOVA or two-way ANOVA, followed by Fisher’s LSD test. Statistical analysis was carried out by GraphPad Prism 8 software (GraphPad Software Inc., San Diego, CA, USA). *P* values less than 0.05 were considered to be statistically significant. All the studies were designed to use randomization and blinding.

## Supplementary information

Supplementary information

Original Data File

## Data Availability

All data that support the findings in this study are available from the corresponding author upon reasonable request.
