# Supplementary material for: GSDME-dependent pyroptosis signaling pathway in diabetic nephropathy
Source: Cell Death Discov. 2023 May 11;9:156. doi: 10.1038/s41420-023-01452-8 (PMC10175547; doi:10.1038/s41420-023-01452-8)
Supplement: Supplementary file 1 — Supplementary information [file 41420_2023_1452_MOESM1_ESM.docx]

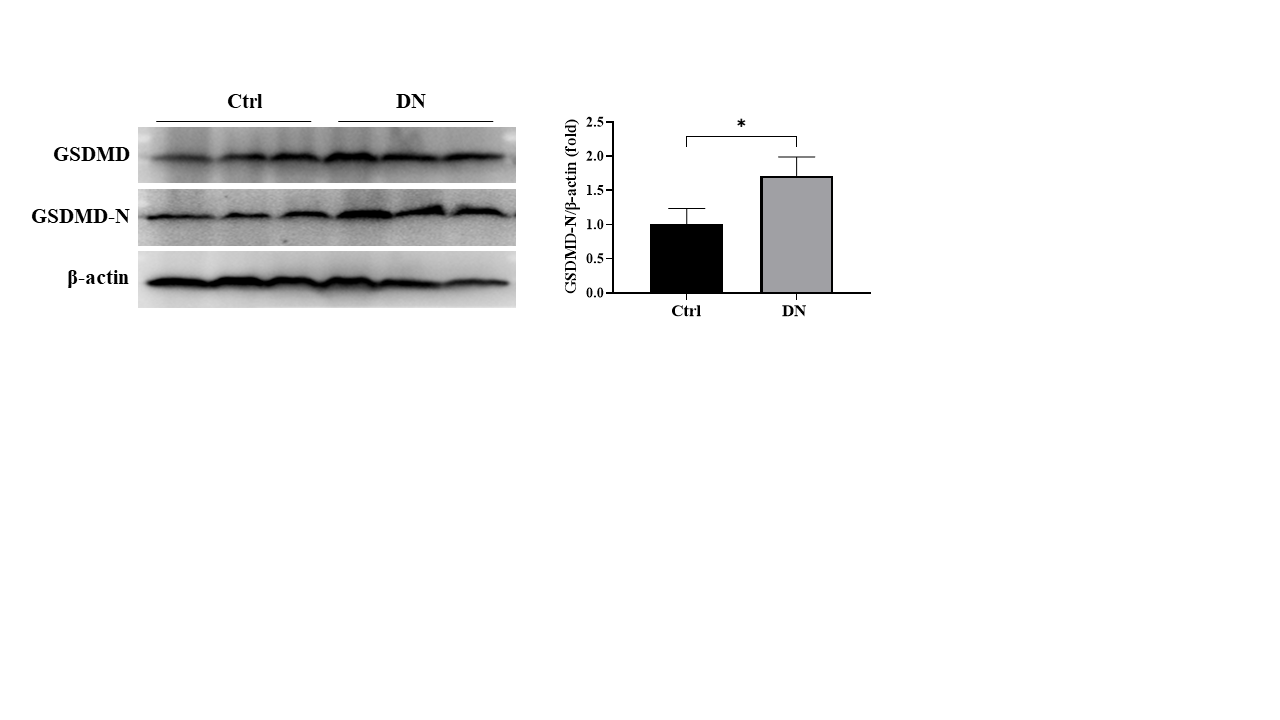


**Figure S1**. **The expression of GSDMD-N was up-regulated in DN rats.** GSDMD and GSDMD-N protein expression were determined by western blotting in rats' kidney cortex at 20 weeks post-DN. Protein level was normalized to the level of β-actin.
