# Supplementary material for: GSDME-dependent pyroptosis signaling pathway in diabetic nephropathy
Source: Cell Death Discov. 2023 May 11;9:156. doi: 10.1038/s41420-023-01452-8 (PMC10175547; doi:10.1038/s41420-023-01452-8)

Figure2

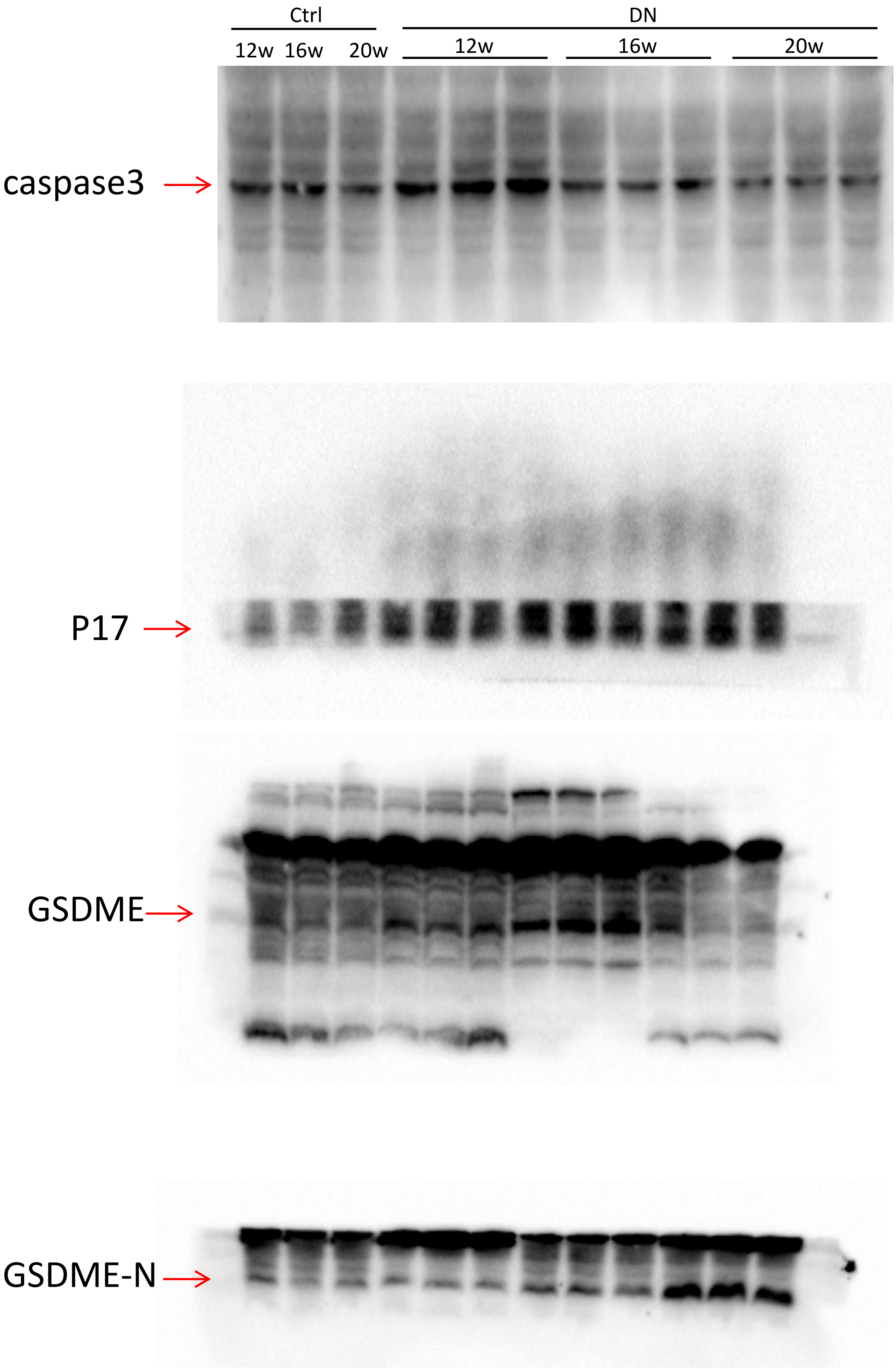

Figure2

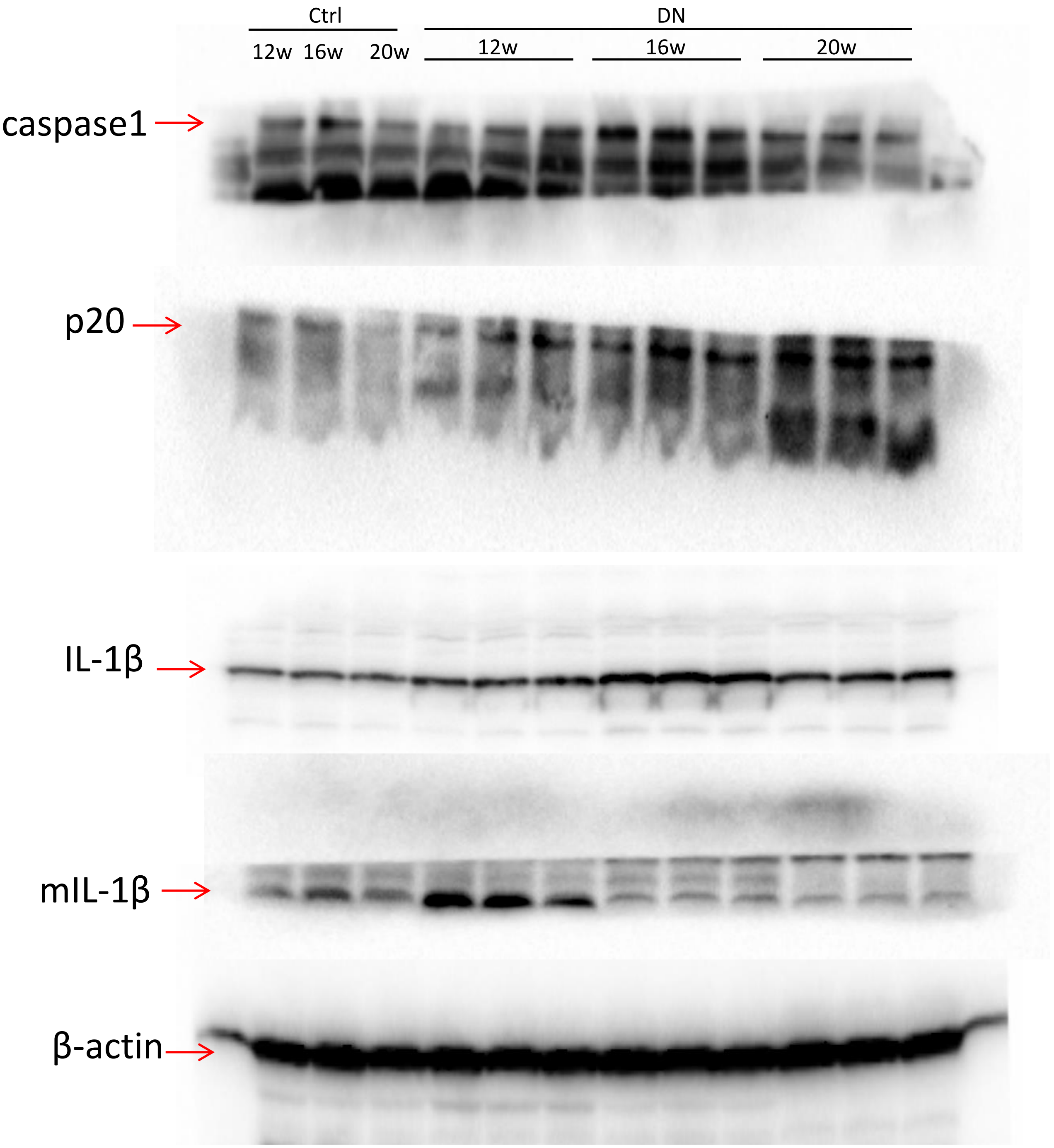
Ctrl
DN
12w 16w 20w
12w
16w
20w
caspase1
p20
IL-1β
mIL-1β
β-actin

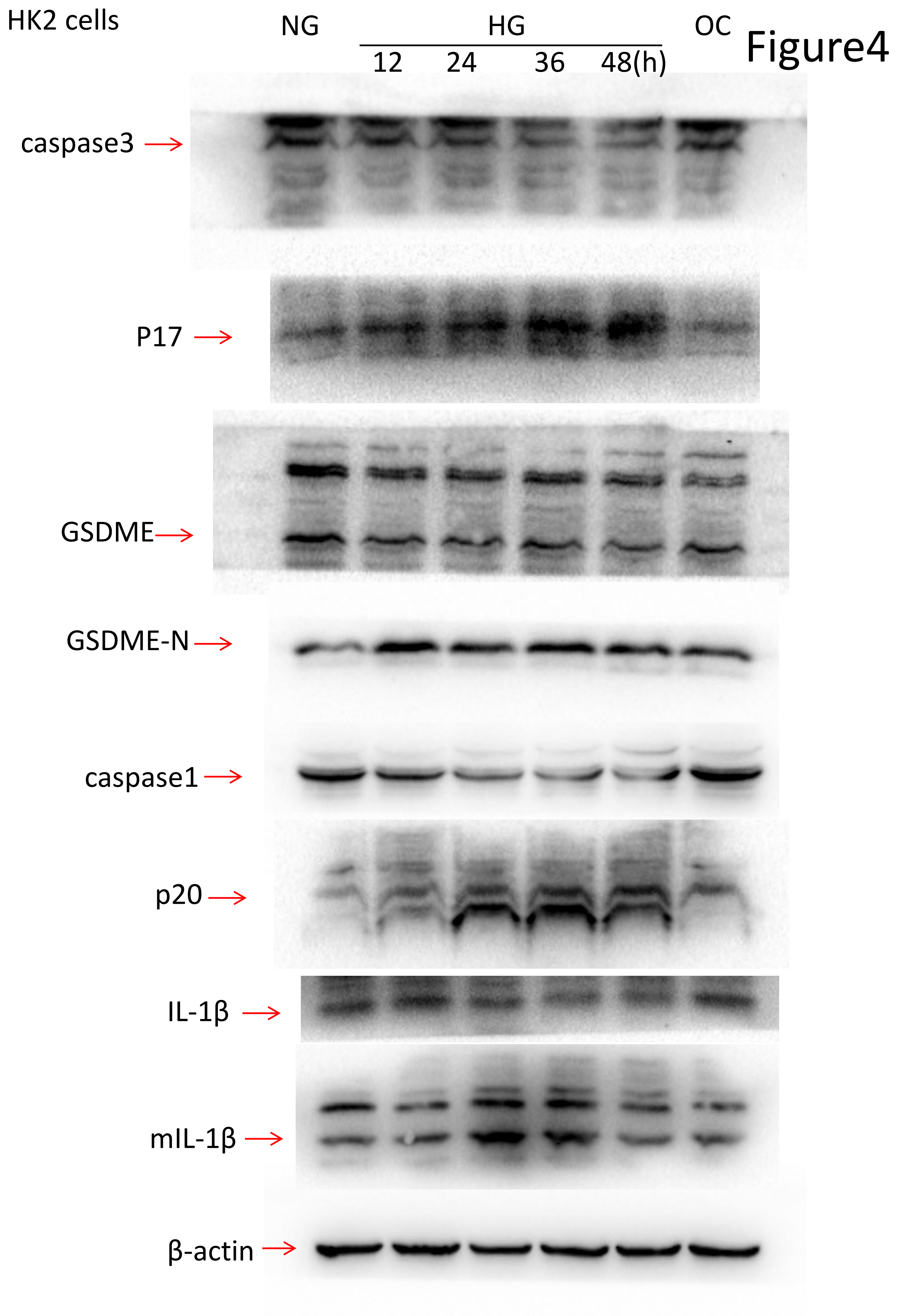
HK2 cells
NG
HG
12
24
36
48(h)
OC
Figure4
caspase3
P17
GSDME
GSDME-N
caspase1
p20
IL-1β
mIL-1β
β-actin

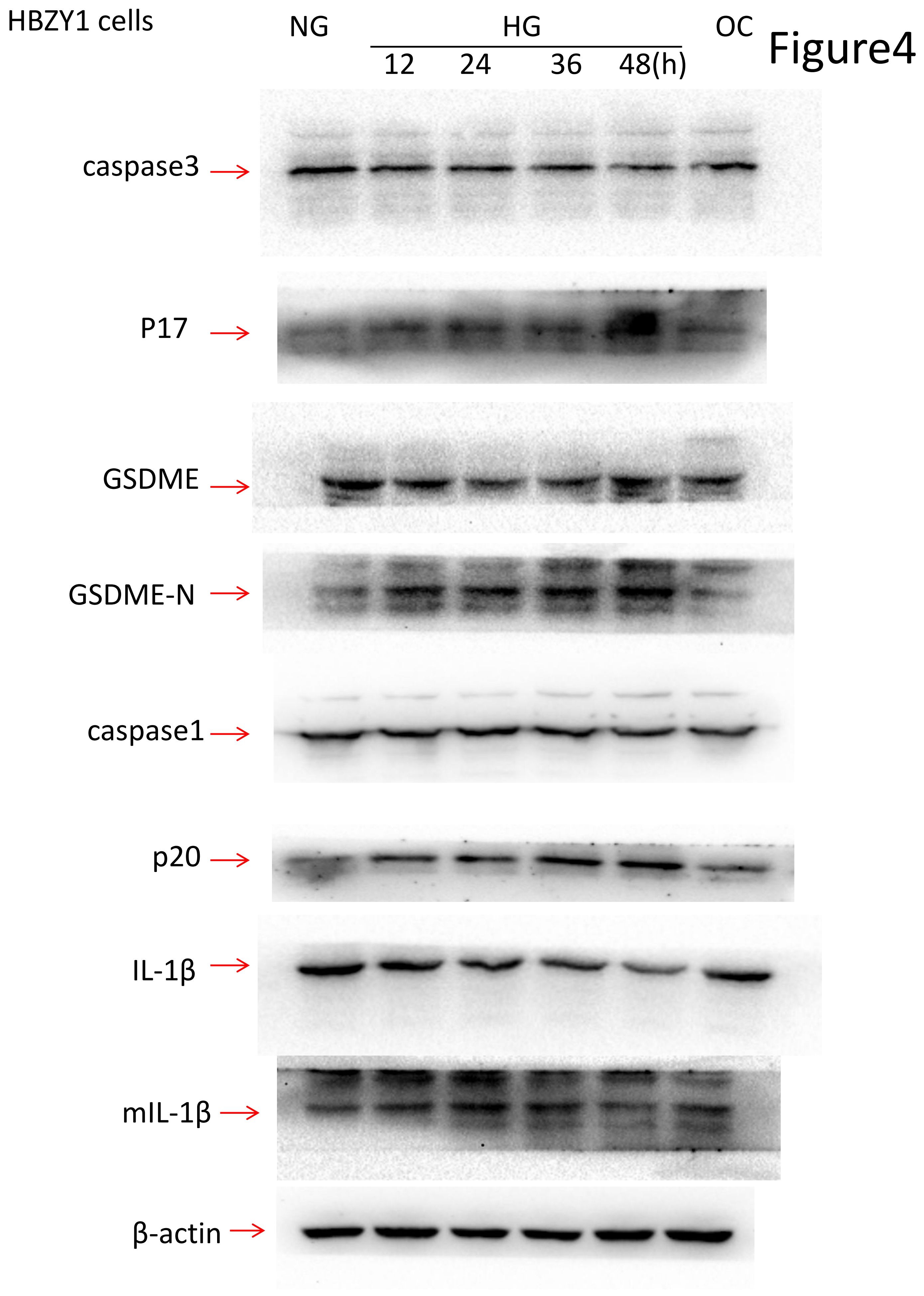
HBZY1 cells
NG
HG
12
24
36
48(h)
OC
Figure4
caspase3
P17
GSDME
GSDME-N
caspase1
p20
IL-1β
mIL-1β
β-actin

Figure5

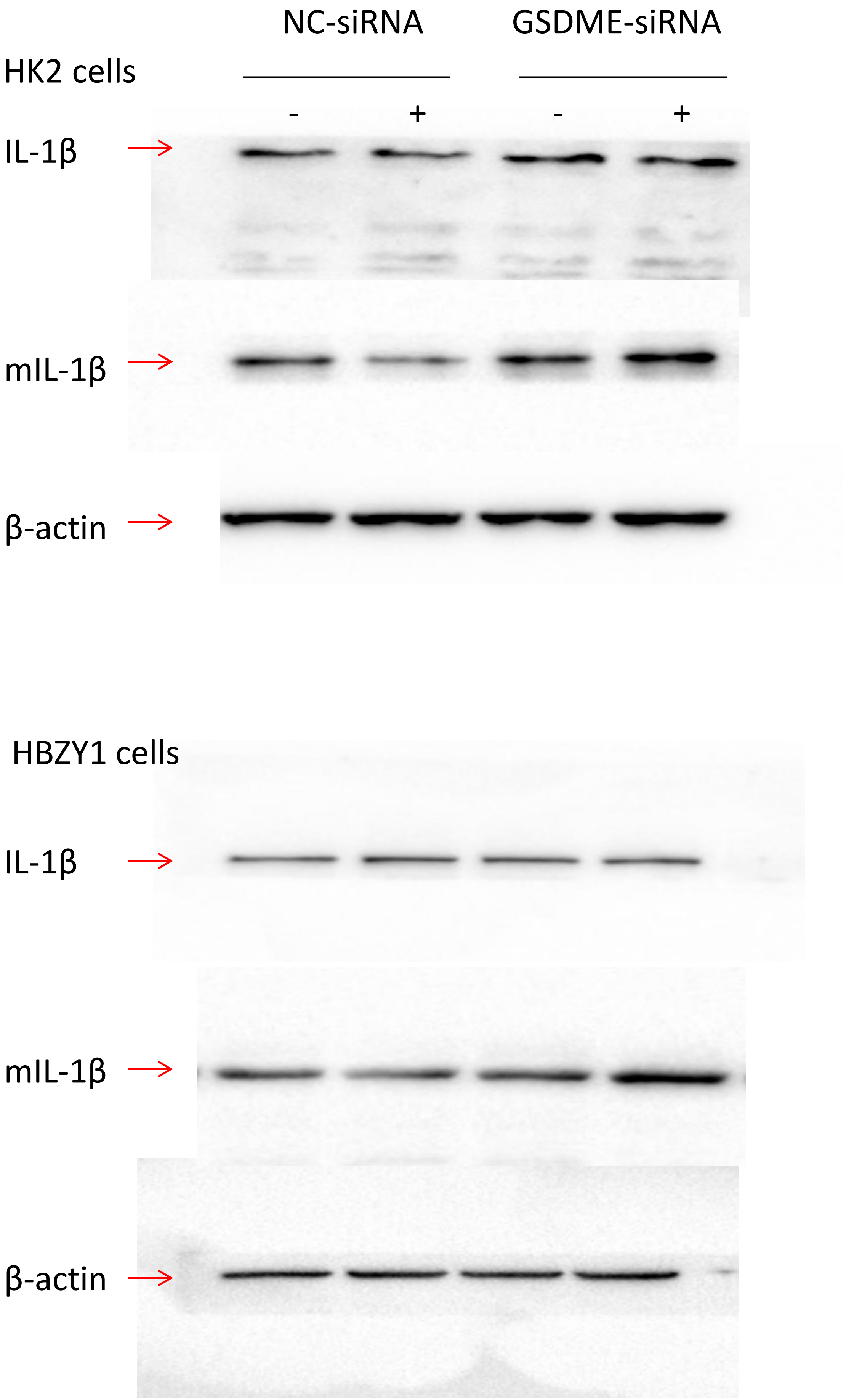

NC-siRNA
GSDME-siRNA
HK2 cells
-
+
-
+
IL-1β
mIL-1β
β-actin
HBZY1 cells
IL-1β
mIL-1β
β-actin

Figure5

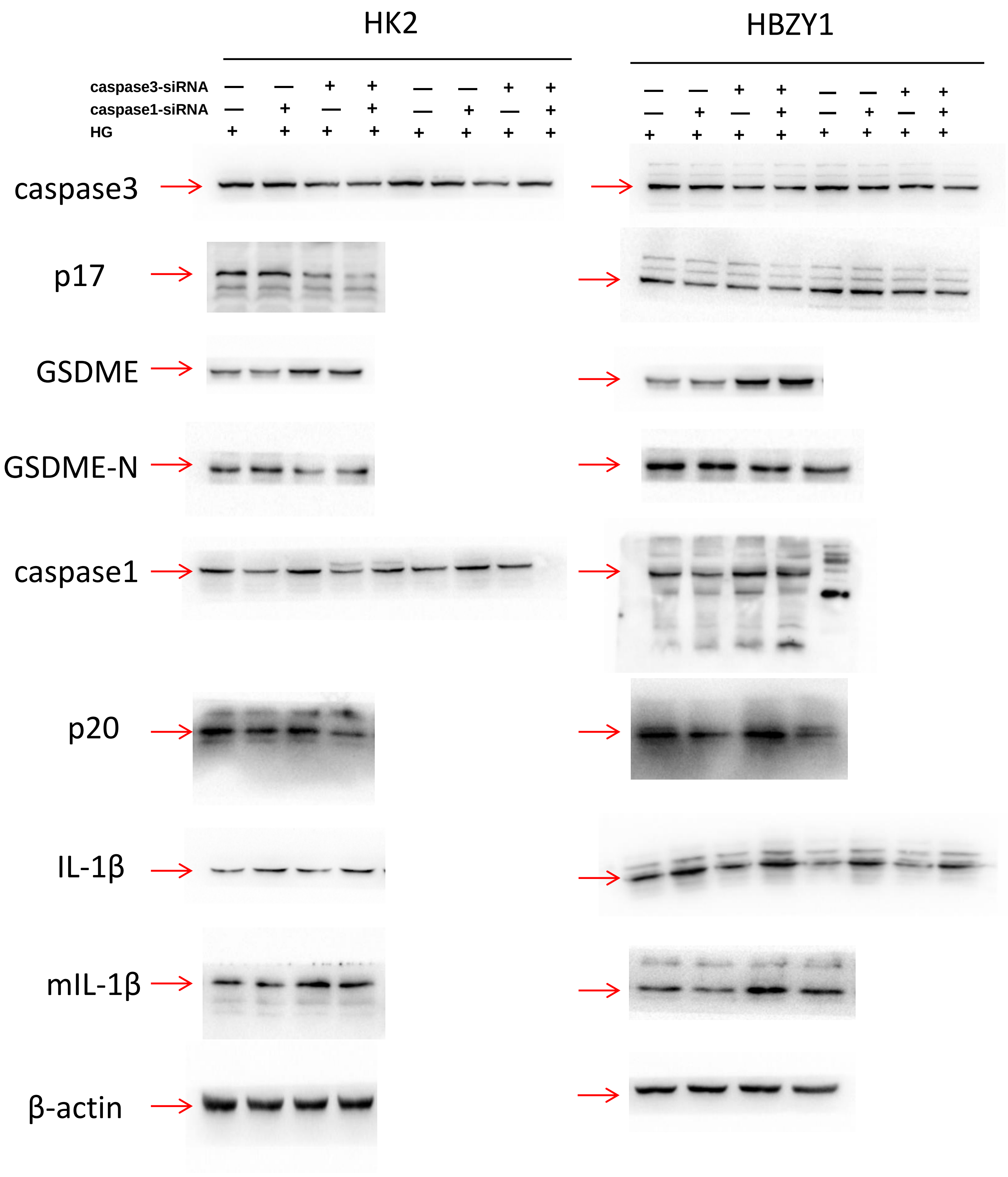

HK2
HBZY1
caspase3-siRNA
caspase1-siRNA
HG
caspase3
p17
GSDME
GSDME-N
caspase1
p20
IL-1β
mIL-1β
β-actin

Figure6

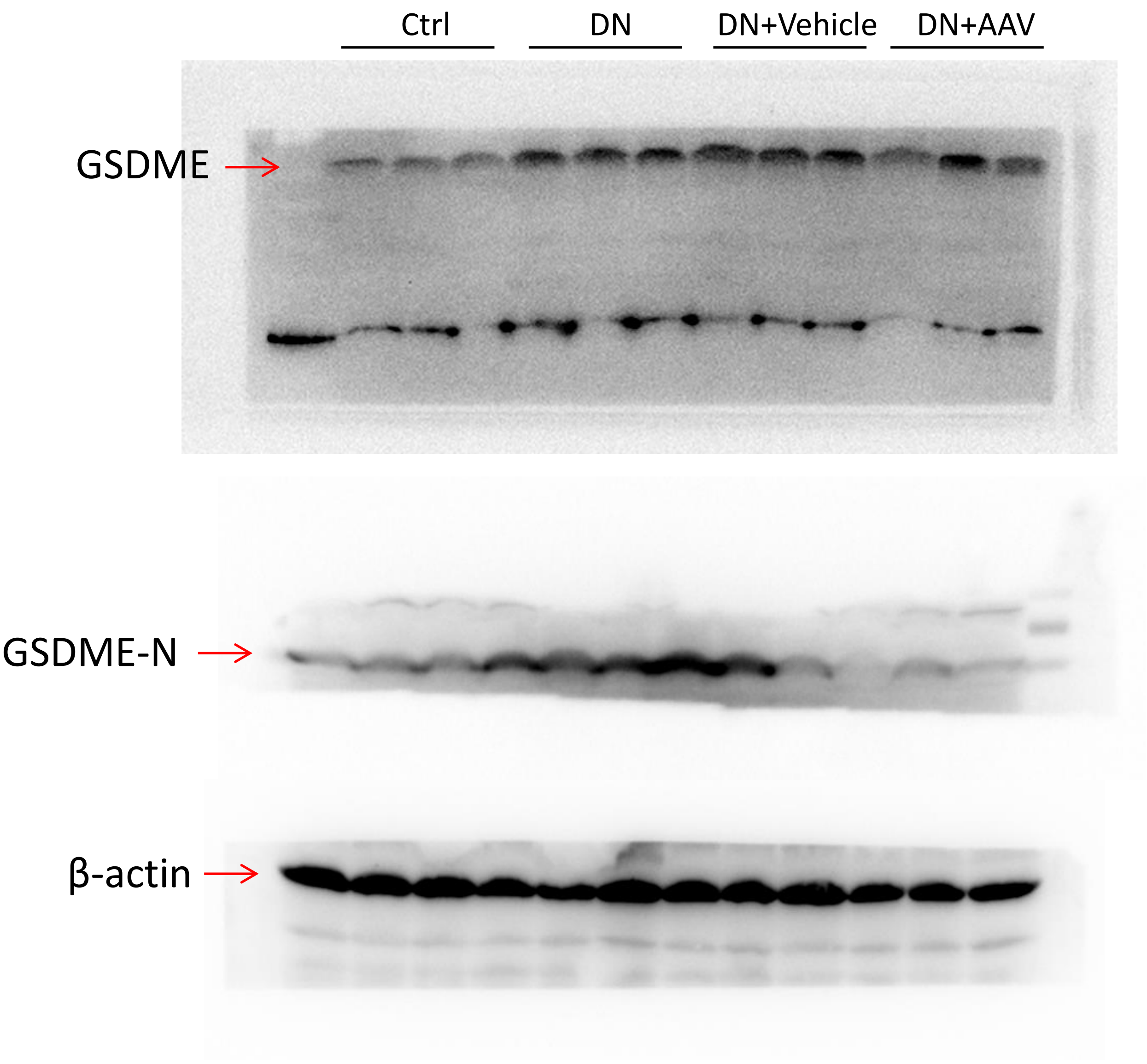

Supplementary Figure 1

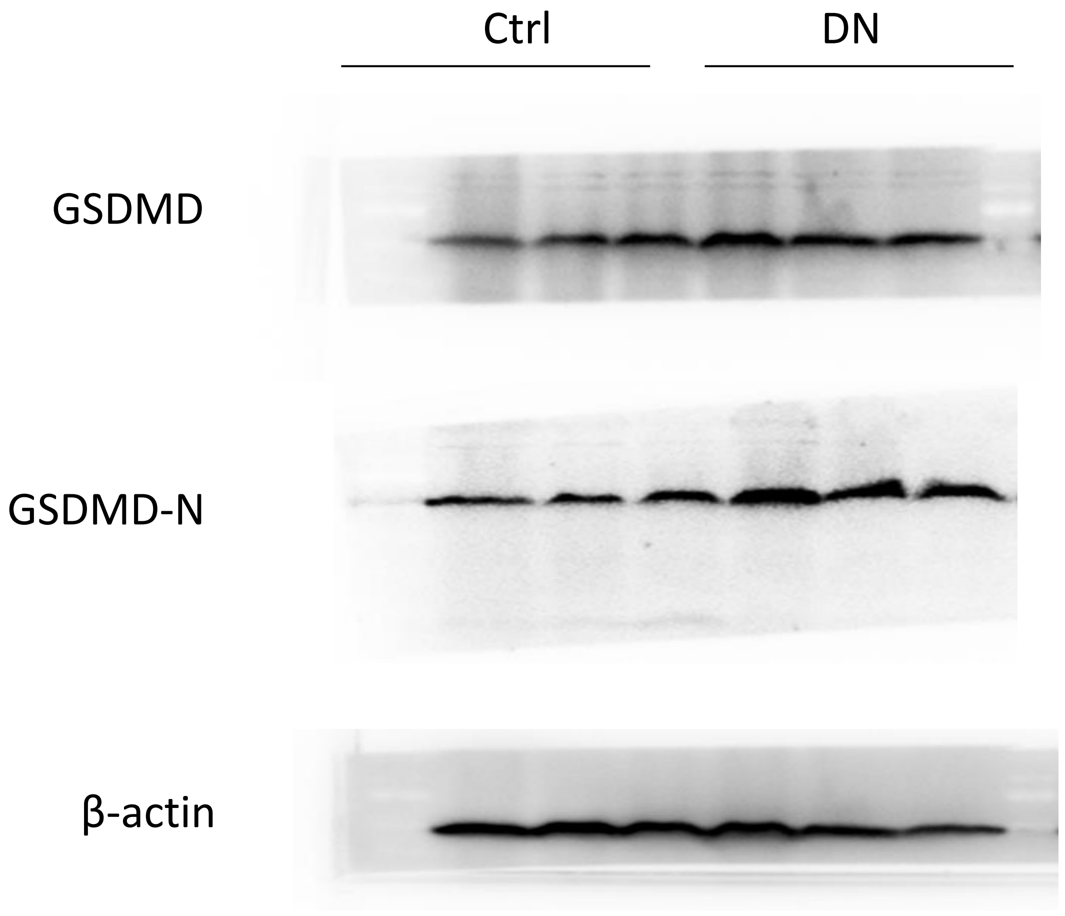

Supplement: Supplementary file 2 — Original Data File [file 41420_2023_1452_MOESM2_ESM.pdf]
